# Experience with local negative pressure (vacuum method) in the treatment of complex wounds

**DOI:** 10.1590/S1516-31802006000300008

**Published:** 2006-05-04

**Authors:** Alexandre Wada, Marcus Castro Ferreira, Paulo Tuma, Gino Arrunátegui

**Keywords:** Wound healing, Vacuum, Plastic surgery, Leg ulcer, Decubitus ulcer, Cicatrização de feridas, Vácuo, Cirurgia plástica, Úlcera da perna, Úlcera de decúbito

## Abstract

**CONTEXT AND OBJECTIVE:**

Use of topical negative pressure over difficult-to-heal wounds has been studied. The objective of this study was to analyze the effects from negative pressure in the treatment of complex wounds.

**DESIGN AND SETTING:**

Case series developed at Hospital das Clínicas, Faculdade de Medicina da Universidade de São Paulo.

**METHODS:**

Twenty-nine patients with complex wounds were treated with the vacuum system and then underwent a surgical procedure to close the wound.

**RESULTS:**

85% of the skin grafts took well, and 87.5% of the local flaps were successful, thus demonstrating adequate wound preparation. The wounds were closed within shorter times than observed using other conventional treatments In two cases, the vacuum system was also used to stabilize the skin grafts over the wounds.

**CONCLUSIONS:**

Use of the vacuum method is safe and efficient for preparing wounds for surgical closure. It allows for an improvement of local wound conditions, and healthy granulation tissue develops with control over local infection.

## RESUMO

**CONTEXTO E OBJETIVO:**

Foi estudado o uso tópico de pressão negativa em feridas de difícil resolução. O objetivo deste estudo foi analisar os efeitos da pressão negativa como tratamento de feridas complexas.

**TIPO DE ESTUDO E LOCAL:**

Série de casos atendidos em um hospital terciário (Hospital das Clínicas – Faculdade de Medicina da Universidade de São Paulo).

**MÉTODOS:**

Vinte e nove pacientes com feridas complexas foram tratados com o sistema a vácuo e, então, submetidos a um procedimento cirúrgico para o fechamento da ferida.

**RESULTADOS:**

85% dos enxertos de pele tiveram boa “pega” e 87,5% dos retalhos locais foram mobilizados com êxito, o que aponta para um preparo adequado do leito da ferida. As feridas foram fechadas em tempo mais curto que o que nós observamos em outros tratamentos convencionais. Em dois casos, o vácuo também foi usado para a estabilização dos enxertos de pele em cima das feridas.

**CONCLUSÕES:**

O vácuo é um método seguro e eficiente no preparo de feridas para o fechamento cirúrgico. Permite melhora das condições locais da ferida, com desenvolvimento de tecido de granulação saudável e controle da infecção local.

## INTRODUCTION

Treatment of wounds is one of the most challenging situations that a plastic surgeon can face. Wound healing consists of a series of events that differ one from the other, leading to uncertainty that frequently makes the surgeon choose incorrect or inadequate methods for treating the problem. Also, the wide range of dressings and drugs that are available for treating chronic wounds means that it is not always clear which of these is most indicated. The surgeon should decide which surgical technique is best indicated for the specific case by considering its effectiveness, safety and cost.

Nowadays, because of greater severity of patients’ illnesses, chronic wounds have become prevalent. In a study conducted in our hospital, about 8% of the patients had chronic wounds, and this situation may be more critical in geriatric hospitals or intensive care units.* The wounds included pressure sores, wounds on diabetic feet, and wounds of vascular origin. The morbidity rate and treatment costs are high and social integration after hospital discharge may be compromised. Recent studies have shown the importance of faster treatment of wounds among in-hospital patients in order to reduce such costs.

The treatment of complex wounds has changed greatly in wound centers over the last decade. It has included aggressive surgical debridement for removal of necrotic tissue, thus allowing better control over local infection. As a second stage, the wound bed should be prepared so as to lead to the formation of healthy granulation tissue and thus reach the third stage: surgical closure using skin grafting, local flaps or microsurgical flap transplantation. Wound contraction and spontaneous epithelization should be kept to a minimum, thus avoiding prolonged hospital stay and recurrence of infection.

New dressings that help in wound preparation have been described, as well as physical methods such as electric current and others. Local negative pressure may be included in this group of agents that improve bed preparation, since this stimulates granulation tissue and provides mechanical drainage of the wound.

The use of negative pressure over the wound surface was first described by Morikwas and Argenta in 1997.^1,2^ It was demonstrated that a vacuum device removing fluid from the wound but not drying it completely produced a better environment for epithelial proliferation and fibroblast migration. Because of this capacity for removing fluids, vacuum conditions also remove bacteria from the wound, thus reducing the bacterial population. Negative pressure applied over the current surface accelerates the formation of granulation tissue, and promotes increased capillary circulation. Moreover, the mechanical effect of the vacuum attracts the borders of the wound towards the center, thus reducing the wound volume.

This method has been approved by the United States Food and Drug Administration (FDA). Because there was no previous experience with this method in Brazil, we started to use it in Hospital das Clínicas, Sao Paulo, in 2002. Preliminary results from three patients were published in 2003^3^ and we now present our more extensive experience, with results from 29 patients.

## OBJECTIVE

The objective of this study was to evaluate the use of negative pressure on the treatment of chronic wounds.

## METHODS

**Figure 1:**
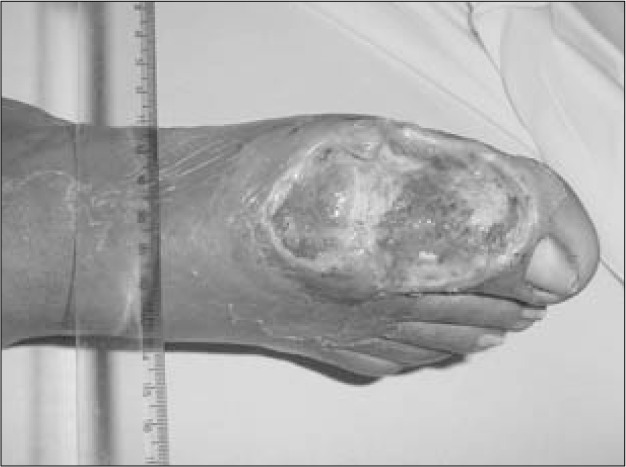
Ulcer on diabetic foot.

Twenty-nine patients with complex wounds were treated in a tertiary hospital (Hospital das Clínicas, Faculdade de Medicina da Universidade de São Paulo), between March 2002 and February 2003. Their mean age was 59.8 years (range: 29-78). Eleven patients were female, and 18 were male. The wound locations were grouped according to topography: 19 in the lower extremities, seven sacral ulcers and three in other locations (one case of dehiscence following abdominoplasty, one dehiscence following breast reconstruction and one traumatic skin avulsion). Among the 19 patients with wounds in the lower extremities, eleven had diabetes (Figure 1), two had vasculitis, and three had wound caused by saphenectomy dehiscence.

The scheme adopted for vacuum use was that it should start forty-eight hours after the necrotic tissue had been surgically debrided.

**Figure 2:**
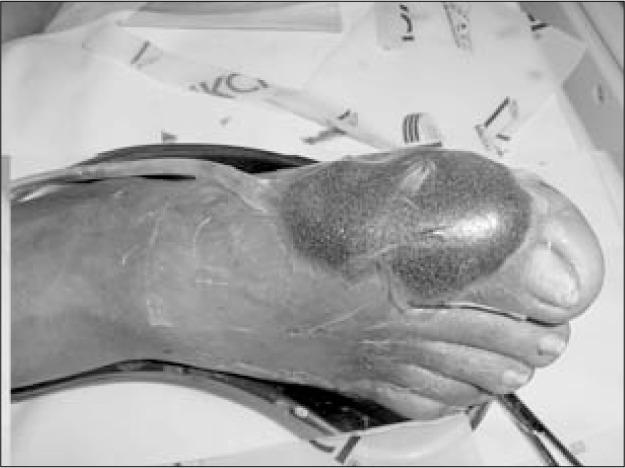
Sponge fitted onto a diabetic foot wound.

**Figure 3:**
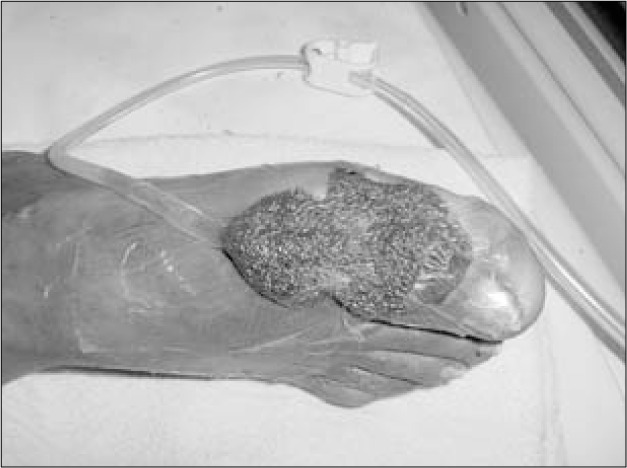
Negative pressure applied to sponge.

The vacuum device (VAC, Kinetic Concepts Inc, USA) consisted of a sponge that was applied directly to the wound, connected to a tube and covered by a transparent adhesive plastic film (Figure 2). The system was linked to a machine that produced negative pressure in a controlled manner (Figure 3). Fluids were collected in a disposable receptacle.

The machine was installed by the plastic surgery staff, and regulated to a 125 mmHg negative pressure, in continuous mode. The sponges were changed every 48 hours, and the reservoir cleared only when completely full. The patients were allowed to stay for a maximum of one hour without negative pressure, during bath time. The device was used for eight days.

**Figure 4:**
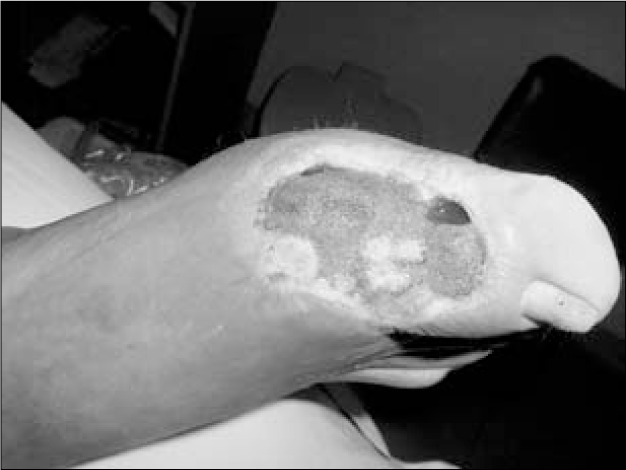
After eight days of negative pressure on diabetic foot.

After an eight-day period with negative pressure (Figure 4), the patients underwent surgical closure of the wound, either using a skin graft or a local flap. In 20 cases, nineteen in the lower extremities and one case following breast reconstruction dehiscence, the use of negative pressure enabled surgical closure using split-thickness skin graft.

**Figure 5:**
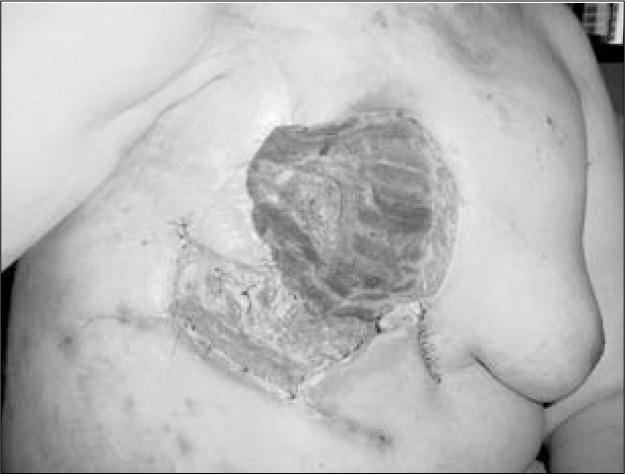
Skin loss after breast reconstruction.

**Figure 6:**
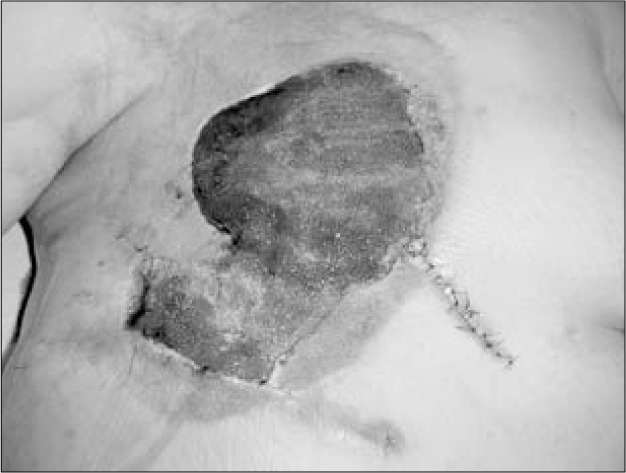
Wound in the breast area after eight days of negative pressure.

In two cases (breast wound and diabetic foot wound), the vacuum method was also used to stabilize the skin graft. The skin graft was meshed 1:1.5, and a gauze was fitted over it. A sponge was used to cover the skin graft and gauze, and film was applied over the system. The vacuum device was installed with 100 mmHg negative pressure, for five days. After this period, it was removed and the skin graft was evaluated (Figures 5 and 6).

In eight patients, all with sacral pressure ulcers, local flap rotation was performed after the eight-day vacuum period.

The results were assessed according to the percentage take of the skin graft, by visual evaluation, or when a local flap was used, according to the percentage survival and wound recurrence.

## RESULTS

One patient with a traumatic skin avulsion from thigh to anterior thorax underwent the vacuum method in order to improve the local and systemic conditions for a skin graft. This patient died on day 6, due to sepsis that was unrelated to the use of the vacuum method, and was the only death during this study.

Among the 20 patients who received skin grafts, there were three partial skin losses (15%) that required new skin grafts in order to achieve complete closure. Minor skin graft losses occurred in five patients (25%), but these patients did not require additional surgical procedures (Table 1). In no case was there wound recurrence. It took an average of 15 days from when surgical debridement was carried out for the wounds to close.

**Table 1.** Patients treated for wound healing with a vacuum device, and surgical outcomes

Patient	Age	Gender	Local	Morbidity	Surgery	Result
MC	47	F	Abdomen	None	Flap	Satisfactory
AMG	58	F	Breast	Hypertension	Skin graft	Satisfactory
ALD	70	M	Lower extremity	Diabetes	Skin graft	Minor graft loss
BS	68	M	Lower extremity	Saphenectomy/coronary disease	Skin graft	Minor graft loss
CAP	55	M	Lower extremity	Diabetes/hypertension	Skin graft	Satisfactory
CED	42	F	Lower extremity	Vasculitis	Skin graft	Satisfactory
CS	49	M	Lower extremity	Diabetes	Skin graft	Minor graft loss
DAO	62	M	Lower extremity	Diabetes	Skin graft	Satisfactory
EBI	61	M	Lower extremity	Hypertension	Skin graft	Satisfactory
ELP	59	M	Lower extremity	Diabetes	Skin graft	Satisfactory
JD	64	M	Lower extremity	Diabetes/hypertension	Skin graft	Reoperated
JI	66	M	Lower extremity	Diabetes	Skin graft	Satisfactory
JSS	78	M	Lower extremity	Diabetes	Skin graft	Satisfactory
LKM	73	F	Lower extremity	Saphenectomy/coronary disease	Skin graft	Reoperated
MJU	52	F	Lower extremity	Vasculitis	Skin graft	Reoperated
MR	48	F	Lower extremity	Hypertension	Skin graft	Minor graft loss
MUI	68	M	Lower extremity	Diabetes	Skin graft	Satisfactory
PFA	29	M	Lower extremity	Hypertension	Skin graft	Satisfactory
RAS	52	M	Lower extremity	Diabetes	Skin graft	Satisfactory
SD	73	M	Lower extremity	Saphenectomy/coronary disease/hypertension	Skin graft	Minor graft loss
UEF	57	M	Lower extremity	Diabetes	Skin graft	Satisfactory
ADJ	75	F	Sacral	Coronary disease	Flap	Satisfactory
AS	36	M	Sacral	Coronary disease/hypertension	Flap	Satisfactory
KIH	73	F	Sacral	Coronary disease/hypertension	Flap	Minor flap necrosis
MLI	76	F	Sacral	Coronary disease/diabetes	Flap	Satisfactory
MOS	71	F	Sacral	Coronary disease/diabetes/hypertension	Flap	Minor flap necrosis
NOS	72	M	Sacral	Coronary disease	Flap	Satisfactory
USL	62	M	Sacral	Coronary disease/hypertension	Flap	Reoperated
FD	39	F	Trunk	None	Died	Died

The eight patients with sacral ulcers achieved wound closure by means of a local fasciocutaneous or myocutaneous flap. There was one case of flap necrosis that required another surgical operation in order to perform flap rotation again. Minor flap losses occurred in two patients, and these only required simple dressing changes in order to achieve total wound closure. The average time required for achieving closure among the patients with flap rotation was 15 days, which was similar to the time required by the skin graft patients.

## DISCUSSION

Local negative pressure is a relative new procedure, but it has already been used in a wide variety of situations, including chronic exudative wounds and some acute wounds. The applications and principles have been studied both experimentally and in the clinical setting. However, there was no previous clinical experience of its use in Brazil.

Hospital das Clínicas is a tertiary medical center and a reference center for the treatment of complex wounds in Brazil. Because of this profile, and the particular characteristics of the public health system in Brazil, complicated wound cases have been seen at an increasing frequency, especially among clinically impaired patients.

Our experience has shown that the vacuum system is an appropriate method for wound management. Complicated cases should not be expected to heal spontaneously by secondary intention. Surgical treatment removes necrotic tissue and the subsequent use of vacuum conditions enables adequate formation of granulation tissue. Thus, the negative pressure system improves local conditions, which is paramount for surgical closure of a wound, and maintains such conditions while the patient's clinical state is stabilized. This allows the surgery to be safer and more predictable, which is critical for a successful outcome.

It is still not completely clear how negative pressure promotes faster and more efficient wound preparation. Continuous drainage of the wound should have a role, since this removes secretions and helps to control infection. Granulation tissue is then able to grow faster, but there may be an additional effect from stimulation of growth factors, induced by controlled ischemia.

In this study, we did not compare the results from the vacuum method with any results from other wound dressings, since the variety of wounds did not allow us to group them as a single category of wounds. However, when these results were compared with our previous experience, it could be seen that the vacuum method allowed the surgical staff to perform the surgical procedures more safely after its introduction. Thus, adequate wound preparation could be achieved, even in clinically compromised patients.

It is important to emphasize the role of the vacuum method in preparing the wound for definitive surgical closure. It can be used to completely close wounds that are not very large. However, the time needed to establish total closure in larger wounds is frequently excessive. Therefore, we accept negative pressure as an auxiliary method for improving the condition of the wound prior to surgical closure, rather than as a definitive method for wound closure.^4^ In our experience, it has helped to reduce hospital stays, thereby reducing the morbidity that is associated with immobilized patients.

By enabling earlier closure, the device might also reduce the total cost of treatment. Its use over skin grafts is another possible advantage of the system.^5,6^ As demonstrated in two cases, the vacuum promoted safe attachment of the skin graft to the wound. In some cases, it may even allow patients to walk while negative pressure is applied.

## CONCLUSIONS

This study demonstrated that this new method was safe, with minimal complications. It was efficient for adequately preparing the wound bed for definite surgical closure, which is an obligatory stage in most cases of complex wounds.
